# Induction of apoptosis by piperine in human cervical adenocarcinoma via ROS mediated mitochondrial pathway and caspase-3 activation

**DOI:** 10.17179/excli2018-1928

**Published:** 2019-03-13

**Authors:** Asif Jafri, Sahabjada Siddiqui, Juhi Rais, Md Sultan Ahmad, Sudhir Kumar, Tabrez Jafar, Mohammad Afzal, Md Arshad

**Affiliations:** 1 Molecular Endocrinology Lab, Department of Zoology, University of Lucknow, Lucknow-226007, India; 2 Molecular and Human Genetics Lab, Department of Zoology, University of Lucknow, Lucknow-226007, India; 3 Department of Biotechnology, Era’s Lucknow Medical College & Hospital, Era University, Lucknow-226003, India; 4 Department of Zoology, Shibli National (PG) College, Azamgarh-276001, India; 5 Department of Molecular Medicine, Era's Lucknow Medical College & Hospital, Era University, Lucknow-226003, India; 6 Department of Zoology, Aligarh Muslim University, Aligarh-202002, India

**Keywords:** anti-tumor, caspase-3, cell cycle kinetics, piperine, ROS

## Abstract

Piperine (1-piperoylpeperdine), a nitrogenous pungent substance, is present in the fruits of black pepper (*Piper nigrum *Linn.) and long pepper (*Piper longum *Linn.). It possesses several pharmacological properties and has been extensively explored for its anti-cancerous activities. The mechanism underlying its anti-cancer potential in human cervical adenocarcinoma (HeLa) cells is not well interpreted. The anti-proliferative effect and the mode of action of piperine were investigated through some potent markers of apoptosis *viz.*reactive oxygen species (ROS) generation, cellular apoptosis and loss of mitochondrial membrane potential (MMP). DNA fragmentation, cell cycle kinetics, caspase-3 activity and cell migration assays were also conducted to observe the efficacy of piperine against HeLa cells. The results showed that piperine exposure induces apoptosis significantly in a dose-dependent manner and inhibits the growth of HeLa cells with an increase in ROS generation, nuclear condensation and delayed wound healing. In addition, piperine also encourages cell death by the loss of MMP, DNA fragmentation and the activation of caspase-3. Growth inhibition of HeLa cells was found to be associated with G2/M phase arrest and sub-G1 accumulation. The present study provides useful insight into the apoptotic potential of piperine and further *in vivo* and clinical studies will be needed for its validation and in the finding of more effective and least toxic regimens against cervical cancer.

## Introduction

Cancer is one of the most common destructive diseases that affect millions of people per year and is the second leading cause of death in humans. Cancer causes approximately 7.6 million deaths every year globally (Prasedya et al., 2016[23]). There is furthermore estimation that the death rates are likely to increase over 11 million in 2030 worldwide (Berrington and Lall, 2012[4]). Among cancer, cervical cancer is the second most common malignancy among women globally; though it is more prevalent in developing countries. Thus, there is a continuous need to search for chemotherapeutic chemicals or naturally occurring drugs to resolve this global health problem. The anticancer therapy can be an effective strategy *i.e.*, inhibiting proliferation and triggering the apoptosis in cancer cells. The present conventional therapies of cancer treatment cause serious side effects, but providentially the plant-derived compounds/phytochemicals were found to possess anti-cancer properties with relatively non-toxic or less toxic effects (Amin et al., 2009[2]).

Black pepper, belonging to the family Piperaceae, is cultivated for fruit and spice and also has been suggested as a reservoir of potential antitumor agents (Lampe, 2003[15]). Piperine possesses multifunctional pharmacological properties such as anti-inflammatory, antioxidant, antidiarrheal, hypolipidemic, hepato-protective, anti-mutagenic, antimicrobial and anti-carcinogenic activities (Srinivasan, 2007[25]; Doucette et al., 2013[7]). Dietary piperine has been shown to increase the therapeutic effect of docetaxel against castration-resistant prostate cancer in xenograft animal models (Makhov et al., 2012[19]). Piperine has inhibited the growth of breast carcinoma by targeting the renewal of cancer stem cells (Kakarala et al., 2010[12]). Furthermore, piperine also possesses anti-apoptotic, anti-oxidative and chemo-protective ability in blastogenesis (Pathak and Khandelwal, 2007[22]). Despite these indirect effects of piperine, there is little known about the anti-cancer effect of piperine on human cervical adenocarcinoma.

In the present study, we have focused on testing the anti-carcinogenic potential of piperine through few potent markers of apoptosis *viz.*, ROS liberation, nuclear condensation, MMP reduction, DNA fragmentation, cell cycle checkpoint, delayed wound healing and caspase-3 activation on human cervical adenocarcinoma, HeLa cells.

## Materials and Methods

### Cell line and culture

Human cervical adenocarcinoma cell line (HeLa) was procured from the cell repository-National Centre for Cell Sciences (NCCS), Pune, India. The cells were cultured in Eagle's minimal essential medium (EMEM) along with 10 % (v/v) fetal bovine serum (FBS) and 1 % antibiotic solution (10,000 U/ml penicillin and 10 mg/ml streptomycin) in a CO_2 _incubator.

### Cell viability assay

MTT reduction assay was performed to evaluate the cell viability of HeLa cells after the treatment of piperine. Approximately, 1×10^4 ^HeLa cells per well were seeded in 96 well cell culture plate in 100 μl EMEM media for 24 h, as per previous protocol (Siddiqui and Arshad, 2014[24]). Treatment with various concentrations *viz.* 10 µM, 25 µM, 50 µM, 100 µM and 200 µM of piperine was performed for 24 h incubation on HeLa cells. After incubation, 20 μl of MTT reagent (5 mg/ml stock) was supplemented in each well and the absorbance was recorded at 540 nm using a microplate reader (BIORAD-680). The cell viability percentage was calculated using the following formula:

Cell viability (%) = [A_540_ (Treated Cells)/ A_540_ (Control Cells)] ×100

### Reactive oxygen species (ROS) generation analysis

The intracellular ROS generation in HeLa cells was performed with the help of fluorescence microscopic imaging after the exposure of different concentrations of piperine at 12 h, using the fluorescence probes 2'-7'-dichlorodihydrofluorescein diacetate (DCFH-DA) as previously reported (Ahamad et al., 2014[1]). The intracellular fluorescence of cells was observed using fluorescence inverted microscope (Nikon ECLIPSE Ti-S, Japan) and images were captured. Further, for the quantitative analysis of ROS, 1×10^4 ^HeLa cells per well were seeded in 96-well black bottom cell culture plate and treated with 25 µM, 50 µM and 100 μM concentrations of piperine. After the treatment period, cells were incubated with DCFH-DA (10 mM) and fluorescence intensity of cells was observed by the multiwell microplate fluorimeter (Synergy Hybrid Multi-Mode Microplate Reader, BioTek) (excitation: 485 nm; emission: 528 nm). The percentage of fluorescence intensity was expressed as the relative fluorescence percentage of treated and control group. 

### Nuclear apoptosis analysis 

The nuclear and chromatin structural alterations in HeLa cells after piperine exposure was examined by using the fluorescent nuclear dye DAPI (4',6-diamidino-2-phenylindole) as per the previous protocol (Ahamad et al., 2014[1]). Apoptotic cells were visualized and quantified under an inverted fluorescent microscope (Nikon ECLIPSE Ti-S, Japan).

### Mitochondrial membrane potential (MMP) assessment 

The decrease in mitochondrial membrane potential (ΔΨm) of HeLa cells after piperine exposure was evaluated by the potentiometric fluorescent dye JC-1 (5,5',6,6'-tetrachloro-1,1',3,3'-tetraethyl benzimidazolecarbocyanine iodide) (Ahamad et al., 2014[1]). The patterns of mitochondrial depolarization were observed with the help of an inverted fluorescent microscope (Nikon ECLIPSE Ti-S, Japan). NIS Elements BR F 4.00.00 imaging software was used to analyze the mitochondrial depolarization patterns, by pairing the red and green fluorescent images to form overlapped color merged images and for cell quantification analysis.

### DNA fragmentation assay 

DNA extraction assay was executed to detect the effect of piperine on HeLa cells according to the previous method (Ahamad et al., 2014[1]). To detect DNA fragmentation, isolated DNA was subjected to electrophoresis on 1.5 % agarose gel containing ethidium bromide and DNA bands were visualized under the ultraviolet illumination gel-doc system (QUANTUMST4-1326.WL/26MX XPRESS, France). 

### Cellular DNA contents by cell cycle analysis

To investigate the distribution of cells at different stages, cell cycle analysis was performed as per the previous protocol (Kumari and Kakkar, 2012[13]). The single cell fluorescence of each nucleus stained with PI dye was measured with the help of flow-cytometer (FACS Calibur, Becton Dickinson, USA). The Data was analyzed using Cell Quest Pro V 3.2.1 software and the result was expressed as a percentage of an overall number of cells in each phase of cell cycle.

### Caspase-3 analysis 

The activation of caspase-3 was performed by using Caspase-3 Colorimetric Kit (Catalog No. K106, BioVision, USA) and immunofluorescence stain following a previous method (Amini et al., 2017[3]). The absorbance was measured at 405 nm with the help of microplate reader (BIORAD-680). For immunofluorescence activity, treated and untreated cells were blocked with the blocking buffer (1 % bovine serum albumin and 0.1 % Tween 20 in PBS) and incubated with rabbit anti-caspase-3 antibody (C8487, SIGMA) at 1:400 dilution followed by Alexa Fluor 594-conjugated secondary anti-mouse caspase-3 antibody (A-11062, Molecular Probe) at 1:400 dilution for 1 h. After incubation, cells were observed under the inverted fluorescent microscope (Nikon ECLIPSE Ti-S, Japan).

### Wound healing analysis

The cell motility inhibition in HeLa cells after treatment of piperine was performed by wound healing assay (Xie et al., 2015[27]). Concisely, HeLa cells were seeded in 12 wells culture plate (2×10^5^ cells/well) for 24 h at 37 °C in 5 % CO_2_ as described earlier. At 90 % confluent cells, a wound area was carefully made in the middle of wells by scratching cell monolayer using a sterile 200 μl micropipette tip. Afterward, the wells were washed three times with PBS to remove any flanking front lines of cells, then treated with 50 µM and 100 µM concentrations of piperine for 0, 24 and 48 h. The width of the wound area was observed at 10-fold magnification using an inverted phase contrast microscope. Image J 1.47v software was used for the width measurements. The wound healing was compared between control cells and the two effective concentrations of piperine by measuring the wound width.

### Statistical analysis 

The data were expressed as a mean ± standard error of the mean (SEM) from three individual independent experiments in triplicates. The differences were calculated by one-way analysis of variance (ANOVA) test followed by the Dunnett's multiple comparison tests with the help of Graph Pad Prism software (Version 5.01). The *p*-value < 0.05 were considered as statistically significant. 

## Results

### Piperine alters morphological changes and anti-proliferative activity in HeLa cells

**Figure 1:**
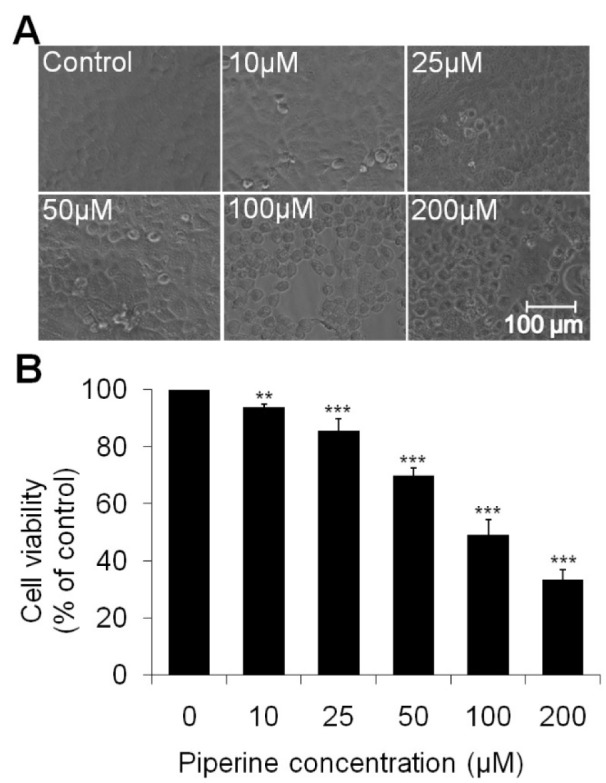
Figure 1. Effect of piperine doses on cell viability in HeLa cells. (A) Cellular morphology of viable and dead HeLa cells after the treatment of 10 μM to 200 μM piperine concentrations. (B) Percentage cell viability was measured by MTT assay after 24 h exposure of piperine on HeLa cells. At least three independent experiments were performed and the values are expressed as means ± SEM, **p < 0.01 and ***p < 0.001 as compared to the control.

Cellular morphological alterations were observed in HeLa cells at different concentrations of piperine treatment under inverted phase contrast microscopy. Photomicrographs of control group exhibit typical morphology of healthy spindle-shaped HeLa cells whereas the shape of piperine treatment groups was largely turned into the round shape. Dose-dependent cellular morphological changes *i.e.,* cellular shrinkage, clusters formation, detachment from surface and decrease in cell density were observed with increase in piperine concentrations (Figure 1A(Fig. 1)).

The anti-proliferative activity of piperine on HeLa cells was estimated by the MTT assay after 24 h of treatment. As evident from Figure 1B(Fig. 1), the viability of HeLa cells at 10 µM concentration of piperine decreases the cell viability about 93.99 % (*p*<0.01) in comparison to the control. However, at the higher concentrations (25 µM, 50 µM, 100 µM and 200 µM) of piperine treatment, showed a significant reduction in viability of HeLa cells, approximately 85.59 %, 69.90 %, 49.27 %, and 33.54 % (*p*<0.001) with respect to untreated cells. The result of cell viability assay suggests that piperine significantly reduces the proliferation of HeLa cells in a concentration-dependent manner. 

### Piperine enhances intracellular ROS and nuclear condensation 

**Figure 2:**
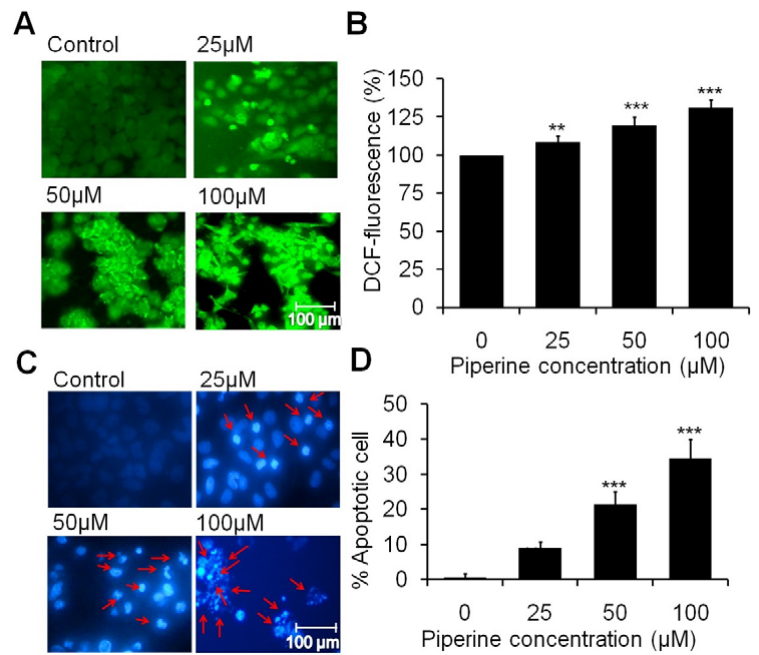
Piperine induces intracellular ROS generation and nuclear condensation in HeLa cells. (A) The photomicrographs show induction in intracellular ROS on HeLa cells at 25 μM, 50 μM and 100 μM concentrations of piperine. (B) Quantitative data represents the percentage of fluorescence intensity on HeLa cells with respect to the control. (C) Photomicrographs exhibits the characteristic fragmented and condensed nuclei (indicated by arrow) in piperine treated HeLa cells. (D) Statistical data expressed as percent of apoptotic cells as compared to the control. Minimum three independent experiments were performed and the values are expressed as means ± SEM, **p < 0.01 and ***p < 0.001 as compared to the control.

The effect of piperine against HeLa cells stimulating the excessive intracellular ROS was observed after 12 h treatment period. Figure 2A(Fig. 2) shows that piperine treatment generates more ROS at 50 µM and 100 µM concentrations in HeLa cells as compared with untreated cells. The quantitative ROS intensity measurement in HeLa cells reveals that 25 µM concentration of piperine enhances the ROS production *i.e*., about 8.53 % (*p*<0.01) with respect to the control. Furthermore, at concentrations of 50 µM and 100 µM of piperine, ROS generation was significantly increased to about 19.62 % and 31.12 % respectively (*p*<0.001) (Figure 2B(Fig. 2)). The result suggested that piperine induces cellular damage and causes cell death by an oxidative stress-mediated pathway. Moreover, as is depicted in Figure 2C(Fig. 2) of nuclear condensation, piperine exhibits dose-dependent nuclear apoptosis in HeLa cells that can be evident by the typical apoptotic features of fragmented nuclear apoptotic bodies and irregular membrane edges. Whereas, untreated cells did not show any noteworthy morphological feature of apoptosis. The quantitative assessment of percent apoptotic cells at 25 µM, 50 µM and 100 µM concentrations of piperine revealed that piperine significantly induces cell death in a dose-dependent manner. At 25 µM concentration, it induces about 9.0 % (*p*<0.01) apoptotic nuclei as compared to control whereas, at 50 µM and 100 µM concentration, the cell death was significantly increased to 21.33 % and 34.66 % (*p*<0.001), respectively (Figure 2D(Fig. 2)). The result concludes that piperine significantly induces condensed and fragmented nuclei in HeLa cells and triggers cell death in an apoptotic manner.

### Piperine alters mitochondrial membrane potential (MMP) of HeLa cells

**Figure 3:**
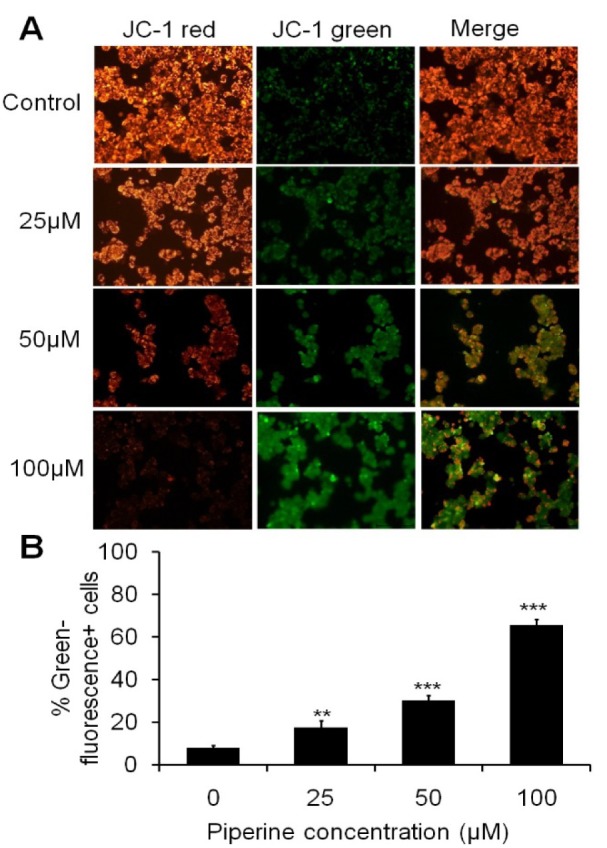
Mitochondrial membrane potential depolarization of HeLa cells treated with different concentrations of piperine stained with JC-1 dye. (A) The photomicrograph shows JC-1 red, JC-1 green and the merged images of HeLa cells treated with 25 μM, 50 μM and 100 μM of piperine. (B) The increase in the intensity of green fluorescence (emitted by green fluorescence positive cells) exhibits a decrease in mitochondrial membrane potential (*ΔΨ*) which represents a characteristic feature of apoptosis. The data is the representative of at least three independent experiments and the values are expressed as means ± SEM, **p < 0.01 and ***p < 0.001 as compared to the control.

Mitochondrial membrane depolarization (*ΔΨ*m) was observed when the cells were treated with piperine and stained with JC-1. The photomicrograph shows the loss of *ΔΨ*min HeLa cells which was evident by the decrease in the intensity of red fluorescence and an increase in the green fluorescence at different effective doses of piperine (Figure 3A(Fig. 3)). An increment in green fluorescence in cells of over imposed images of JC-1 red and JC-1 green shows the potent apoptotic activity of piperine. The enhancement of green-fluorescence^+^ in HeLa cells was found to be approximately 17.33 %, 30.33 % and 65.66 %, for doses 25 μM, 50 μM and 100 μM respectively (Figure 3B(Fig. 3)). The MMP depolarization result suggested that piperine significantly reduces mitochondrial membrane potential of HeLa cells and thus induces cellular apoptosis. 

### Piperine induces apoptotic markers of DNA fragmentation, cell cycle arrest, and caspase-3 

**Figure 4:**
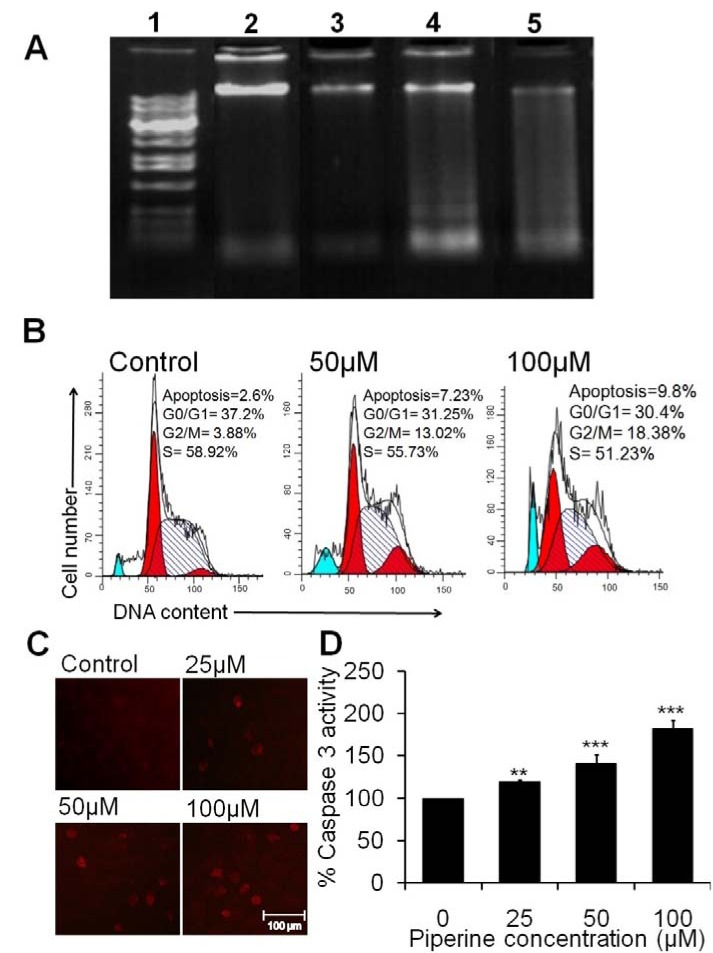
Piperine stimulates DNA fragmentation, cell cycle arrest and caspase-3 activation in cervical carcinoma HeLa cells. (A) Photograph of agarose gel under UV light, exhibiting fragmented DNA of HeLa cells treated with 25 μM, 50 μM and 100 μM of piperine. Lane 1: showing 1 kb DNA marker; Lane 2: untreated HeLa cells (control); Lane 3: HeLa cells treated with 25 μM of piperine; Lane 4: HeLa cells treated with 50 μM of piperine and Lane 5: HeLa cells treated with 100 μM of piperine. (B) The photomicrograph displays the apoptosis and phase distribution of the cell population in HeLa cells at 50 μM and 100 μM concentrations of piperine measured by flow cytometry. (C) Photomicrograph shows immunofluorescence staining of HeLa cells with the caspase-3 immunofluorescence stain. (D) Quantitative data were measured by caspase-3 colorimetric assay kit and the values are expressed as means ± SEM of at least three independent experiments, ***p < 0.001 as compared to the control.

As is depicted in Figure 4A(Fig. 4) of DNA fragmentation data, the control cells (without piperine exposure) show clear bands of intact or undamaged chromosomal DNA, a slight laddering was observed at 25 μM of piperine treatment, however, an increased DNA destruction and fragmentation were observed at 50 μM and 100 μM of piperine on the agarose gel electrophoresis. In cell cycle analysis, the sub-diploid cells in comparison to control cells were calculated for the estimation of the apoptotic cells on cell cycle histogram. As is evident from Figure 4B(Fig. 4), piperine at 50 μM exposure induces 7.23 % apoptotic cells as compared to control. At 100 μM concentration of piperine, apoptotic cells were increased to 9.8 %. The percentage of cells in G2/M phase in control group was found to be 3.88 %, while at 50 μM and 100 μM concentration of piperine, the cell cycle checkpoint remarkably increased to 13.02 % and 18.38 %, respectively.

While the subG1 phase (an indication of apoptosis) was found to be 2.6 % at control group, however, it was increased to 7.23 % and 9.8 % at 50 μM and 100 μM of piperine, respectively. Interestingly, the S phase of the cell cycle, the cell population was also reduced in a dose-dependent manner in piperine treated HeLa cells. These results have evidently supported that piperine induces cellular apoptosis followed by cell cycle arrest at the G2/M checkpoint and by increasing in the subG1 phase of cell cycle in HeLa cells. Moreover, the effect of piperine was further investigated to determine whether the apoptosis was induced by caspase-3, a key apoptotic signaling molecule. As revealed by Figure 4D(Fig. 4), piperine significantly (***p <0.001) encouraged the activation of caspase-3 approximately 19.55 %, 41.65 % and 82.94 % at 25 μM, 50 μM and 100 μM concentrations in HeLa cells. Figure 4C(Fig. 4) exhibits the distribution of cells detected by the activation of caspase-3 antibodies in HeLa cells.

### Piperine delays wound healing and cell motility in HeLa cells

**Figure 5:**
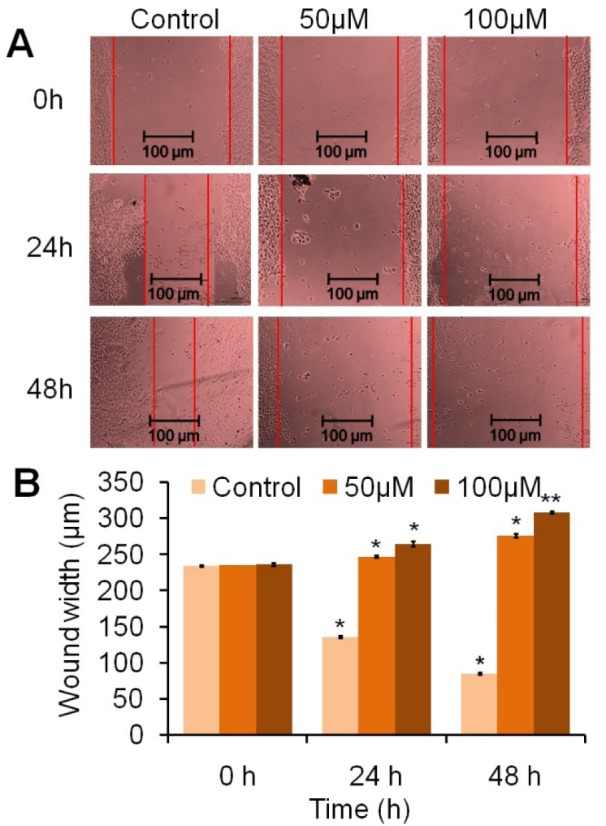
Piperine hinders the wound healing responses against HeLa cells. (A) HeLa cells were treated with 50 μM and 100 μM concentration of piperine and photographed at 0, 24, and 48 h by a phase contrast microscope at 10X magnification. (B) Quantitative data were represented as wound width *vs* time (h). Piperine (50 and 100 μM) significantly delayed wound healing and proliferation in the HeLa cells at 24 and 48 h when compared to the untreated cells. Values are expressed as means ± SEM of at least three independent experiments, *p < 0.05 and **p < 0.01 as compared to the control.

Piperine hinders cell proliferation along with cell motility of HeLa cells in a time and concentration-dependent manner (Figure 5(Fig. 5)). Untreated HeLa cells showed a time-dependent wound healing ability by forming colonies migrated into the scratched area (Figure 5A(Fig. 5)). As compared to control cells, the cells treated with 50 μM and 100 µM concentrations of piperine displayed a wider wound area at 24 and 48 h after the wound generation, indicating a defect in migration. As compared to the control following 24 h, the gap widths in the piperine-treated cells were 247.45 and 265 µm following treatment with 50 μM and 100 μM, respectively (Figure 5B(Fig. 5)). Similarly, at 48 h of the treatment, the gap widths in the piperine-treated cells were 276.5 and 308.5 µm as compared to control. Thus, piperine-treated HeLa cells displayed an approximately 1.5-fold wider wound width after 48 h, as compared to the control group. The result reveals that piperine impairs the cell motility and invasion ability of HeLa cells, which suggests the anti-proliferative and anti-invasive property of piperine in human cervical carcinoma cells.

## Discussion

Piperine, an alkaloid present in black pepper, significantly inhibits the carcinoma of cervix *i.e.,* checks HeLa cell proliferation. Various studies evidenced that piperine significantly reduced the growth of human cancer cells (Yaffe et al., 2013[28]; Greenshields et al., 2015[10]). However, the anti-proliferative action of piperine reducing MMP, cell cycle inhibition and caspase-3 activation against cervical cancer is still to be investigated. Our results demonstrated that piperine possesses significant cytotoxic effects against HeLa cells in a dose-dependent manner. 

Induction of apoptosis in tumorous cells is considered to be a valuable process in the treatment of various cancers. Apoptosis is characterized by the typical morphological variation in the cells *viz.* cell shrinkage, nuclear chromatin condensation and formation of fragmented apoptotic bodies (Evan and Vousden, 2001[8]). In the present study, significant unique apoptotic morphology was observed after exposure of piperine at various concentrations against HeLa cells (Figure 1(Fig. 1)). 

A study by Tawani et al. (2016[26]) has reported the cytotoxic effects of piperine against four different human cancer cell lines *viz.* breast carcinoma (MCF-7), liver carcinoma (HepG2), cervical carcinoma (HeLa) and prostate cancer cells (PC3) by using MTT assay. The result of this cytotoxic cell death was found to be in agreement with the present study of piperine. However, in the present study, the apoptotic potential of piperine was further tested by ROS measurement, MMP reduction, cell cycle arrest and caspase-3 activation in HeLa cells.

The present study has investigated that piperine successfully induces ROS generation and indirectly induces cellular apoptosis (Figure 2A and 2B(Fig. 2)). Too much ROS production induces oxidation of biomolecules, which results in mitochondrial DNA mutations, aging, and cell death (Circu and Aw, 2010[6]). Therefore, increased intracellular ROS production can implicate cellular damage of HeLa cells. Interestingly, a previous study also supported our results that ROS is the crucial agent responsible for oxidative DNA damage and cell death during oxidative stress (Chernyak et al., 2006[5]).

Moreover, the present study has also examined the effect of piperine on MMP level, which regulates mitochondrial apoptosis. MMP plays a crucial role in regulating cellular apoptosis (Lee and Wei, 2000[16]). In a previous study, no changes were found in Bax and Bcl-2 protein in 4T1 cells, which suggest that the apoptosis induced by piperine is independent of the Bcl-2 pathway (Lai et al., 2012[14]). In addition, the cell cycle data has demonstrated that piperine inhibits the proliferation of HeLa cells due to the induction of cell cycle arrest. Since the induction of apoptosis and cell cycle arrest are the major targets for cancer chemotherapy (Gerl and Vaux, 2005[9]). The cell cycle data of HeLa cells has revealed that piperine treatment arrests the cell at G2/M phase and consequently leads to cell apoptosis. A recent study has reported that the piperine nanofibers encouraged apoptosis *via* augmenting ROS and arresting the cell cycle in G2/M phase in HeLa cells (Jain et al., 2016[11]). Interestingly, the present finding has also stated the same mode of action of piperine against HeLa cells. Another study has reported that pipernonaline, a piperine derivate from *P. Longum* Linn., induces G0/G1 phase arrest in human prostate cancer cells (Lee et al., 2013[17]). A previous study has suggested that piperine induces p21Cip1 protein and inhibits cyclins which might be accountable for the induction of cell cycle arrest (Ouyang et al., 2013[21]).

Caspase-3 is the main downstream caspase in the apoptotic pathway and hence we compared its activity in treated and untreated control cells. The results from both *in vitro* a colorimetric assay and immunofluorescence staining assay have revealed that piperine significantly induces the caspase-3 level in HeLa cells (Figure 4C and 4D(Fig. 4)). The caspase-dependent pathway is the key apoptotic pathway for cellular apoptosis which cleaves the nuclear DNA of apoptotic cells (Lee et al., 2004[18]). A previous study has reported that the antitumor effect of piperine on mouse 4T1 mammary carcinoma was probably by increasing the caspase-3 and decreasing the expression of cyclin B1 (Lai et al., 2012[14]). Interestingly, our study has also supported that piperine induces apoptosis in HeLa cells *via *a caspase-dependent pathway. 

Additionally, piperine compound has been found to inhibit the migration of human cervical cells in both concentration and time-dependent manner. Since both cell migration and invasion have a decisive role in the dissemination of cancer cells and metastases. Cell migration is one of the important steps during the proliferative phase of wound healing (Martin et al., 2013[20]). Compared with untreated cells, the migration rate of treated cells may be due to cytotoxicity of piperine at high concentration.

In conclusion, the present study has revealed that piperine inhibits the proliferation of HeLa cells by reducing cell viability, ROS increment, MMP depolarization, inducing nuclear condensation, cell shrinkage and DNA fragmentation. Also, piperine has suppressed the growth of cervical carcinoma by inhibiting wound healing, cell cycle blockage and activation of caspase-3. Further studies will be needed to determine the underlying antitumor action of piperine at *in vivo* level and clinical study is required to develop anticancer drugs for cervical cancer therapy.

## Notes

Asif Jafri and Sahabjada Siddiqui contributed equally to this work.

Sahabjada Siddiqui, Md Sultan Ahmad (Department of Zoology, Shibli National (PG) College, Azamgarh-276001, India; Tel.: +91-522-2370813, Fax: +91-522-2740230, E-mail: sultansnc@gmail.com) and Md Arshad (Molecular Endocrinology Lab, Department of Zoology, University of Lucknow, Lucknow-226007, India; Tel.: +91-522-2370813, Fax: +91-522-2740230, E-mail: arshadm123@rediffmail.com) contributed equally as corresponding authors.

## Acknowledgements

Thanks are due to the Uttar Pradesh Council of Science and Technology (UPCST) File No. CST/SERPD/D-299 and University Grants Commission (UGC) 42-500/2013SR in the form of a research grant. Asif Jafri is thankful to Council of Scientific and Industrial Research (CSIR), India for Senior Research Fellowship (File No. 09/107 (0393)/2018- EMR-I) and Sahabjada Siddiqui is thankful for the research facility of the Department of Biotechnology, Era’s Lucknow Medical College & Hospital, Era University, Lucknow, India. Authors are also thankful to DST-PURSE and UGC-SAP for providing a grant to central instrumentation facilities to the Department of Zoology, University of Lucknow for smooth conduction of the experiments.

## Conflict of interest

The authors declare that there are no conflicts of interest.
